# Clinical utility of neurofilament light chain as a biomarker for disease onset and progression in hereditary transthyretin amyloidosis

**DOI:** 10.3389/fneur.2025.1660344

**Published:** 2025-10-08

**Authors:** Álvaro Gragera-Martínez, Cristina Borrachero Garro, Francisco Muñoz Beamud, Ana Manovel Sánchez, Andrés González Macías, Mariano Pizarro Sánchez, Amelia Jiménez Heffernan, Ana Serrano Mira, Beatriz Macías Dominguez, Sandra Garcia Garrido

**Affiliations:** ^1^Genetic Unit, Department of Clinical Chemistry, Juan Ramon Jimenez University Hospital, Huelva, Spain; ^2^Multidisciplinary Amyloidosis Unit, Juan Ramon Jimenez University Hospital, Huelva, Spain; ^3^Department of Internal Medicine, Juan Ramon Jimenez University Hospital, Huelva, Spain; ^4^Department of Cardiology, Juan Ramon Jimenez University Hospital, Huelva, Spain; ^5^Department of Neurophisiology, Juan Ramon Jimenez University Hospital, Huelva, Spain; ^6^Department of Nuclear Medicine, Juan Ramon Jimenez University Hospital, Huelva, Spain; ^7^FABIS Fundacion, Juan Ramon Jimenez University Hospital, Huelva, Spain

**Keywords:** neurofilament, ELISA, SiMoA, transthyretin, amyloidosis ATTRv

## Abstract

**Background:**

Neurofilament light chain levels (NfL) have emerged as a biomarker for early diagnosis and follow-up of hereditary transthyretin variant amyloidosis (ATTRv). We evaluated the most accurate technique for NfL quantifying in ATTRv healthy carriers and symptomatic patients in real-life practice, and assessed whether NfL may represent a reliable biomarker of disease onset and progression.

**Methods:**

Serum NfL were measured using ELISA and the single-molecule array (SIMoA) technique. Disease severity was assessed with a polyneuropathy disability score (PND).

**Results:**

Seventy-five subjects with pathogenic transthyretin variant (40 ATTRv healthy carriers and 35 ATTRv patients) were enrolled. We observed a significant correlation between ELISA and SIMoA assay (Pearson’s R2-value = 0.9899). Compared to healthy carriers, patients with symptomatic ATTRv had statistically higher serum NfL levels (*p* < 0.001). We propose a NfL cut-off of 7.9 pg./mL to distinguish between healthy carriers and ATTRv patients with high diagnostic accuracy (AUC = 0.847; *p* < 0.001; sensitivity = 90.0%; specificity = 55.0%), whereas the NfL threshold of 18.4 pg./mL discriminated the transition from patients with PND I to PND ≥ II (AUC = 0.695; *p* < 0.001; sensitivity = 67.0%, specificity = 86%).

**Conclusion:**

Serum NfL can be accurately quantified using both ELISA and SIMoA array, and it seems to be a reliable biomarker to detect the transition from presymptomatic to symptomatic disease onset and to monitor disease progression.

## 1 Introduction

Hereditary transthyretin variant amyloidosis (ATTRv), also known as Andrade’s disease or familial amyloid polyneuropathy, is a rare, highly debilitating, autosomal-dominant neurological disorder characterized by a progressive peripheral sensorimotor and/or autonomic neuropathy (1, 2). ATTRv exhibits an adult-onset pattern, with variable and incomplete penetrance, and a progressive and life-threatening complications if left untreated and the mean duration from the disease onset to death of 7.3 years (3). Despite the global presence of ATTRv, its principal endemic regions are Portugal, Japan, Sweden and Brazil (4, 5). In Spain, there are two endemic foci, the largest of which is on the island of Mallorca, and the other near Portugal, in Valverde del Camino (Huelva province) (6). In Spain and Portugal, ATTRv amyloidosis is typically caused by mutations in the TTR gene encoding transthyretin protein (TTR), commonly being the p. Val50Met variant mutation (6). These mutations lead to the accumulation of misfolded TTR proteins. The extracellular deposition of mutant TTR as insoluble amyloid fibrils in multiple organs leads to progressive neurological disorders (7, 8). The peripheral and autonomic nervous system, as well as the heart, are commonly involved organs. However, other organs, such as the eyes, kidneys, and gastrointestinal tract, may also be affected, resulting in a life-threatening multisystemic disease (9, 10). Therefore, early and accurate diagnosis of ATTRv represents one of the major challenges (11). The current diagnosis of the disease is based on clinical management and nerve conduction tests, which are invasive for the patient and take a long time to see changes with clinical consequences (1).

Until very recently, liver transplantation was the treatment of choice for patients with ATTRv (12). Fortunately, in recent years, the management of ATTRv has undergone a significant transformation, going from incurable to treatable owing to the advancements in disease-modifying targeted therapies. These therapies include TTR-stabilizers, which prevent the dissociation of the TTR protein into amyloidogenic monomers, and TTR gene silencers, which specifically inhibit the hepatic synthesis of TTR, possessing the capability to significantly suppress TTR production (13–15). The prognosis for ATTRv patients has improved due to the development of those novel therapies, highlighting the significance of prompt diagnosis and treatment initiation (10). Nowadays, those drugs represent a backbone therapy for patients with ATTRv, especially in the early stages of the disease when they can prevent or delay the onset of more severe stages (16). Hence, any delay in a timely diagnosis may represent a major obstacle to the optimal management and prognosis of patients with ATTRv. Moreover, presymptomatic testing and management of TTR healthy carriers also remain an important consideration in ATTRv care (17). Therefore, the need for reliable disease biomarkers is gaining further attention, both for patients with symptoms and to identify disease in presymptomatic or early disease stages. Biomarkers are particularly needed across endemic areas, where ATTRv carriers require close monitoring to detect clinical symptoms as early as possible, potentially leading to a better quality of life in the future.

Recent research has focused on finding an early, non-invasive and sensitive biomarker for in patients with ATTRv (18). Several investigations have shown that neurofilament light chain (NfL) levels in ATTRv patients are related to an increase in axonal damage and positively correlated with disease progression and response to different treatments, supporting the use of NfL levels in screening asymptomatic healthy carriers, disease progression monitoring, and treatment effect (4, 19–22). Consequently, NfL has emerged as a most promising biomarker for ATTRv, which may enable early diagnosis and facilitate regular disease monitoring (2, 9, 22–25).

In this study, we aimed to establish the most accurate technique for quantifying NfL in serum of ATTRv healthy carriers and ATTRv patients using two analytical platforms. Additionally, our objectives were to evaluate whether serum NfL levels in patients with ATTRv are an accurate diagnostic and prognostic biomarker that can detect the transition of presymptomatic ATTRv carriers to the onset of symptomatic disease. Furthermore, we aimed to determine its reliability in correlating with disease staging and its utility in guiding the evaluation of disease progression.

## 2 Materials and methods

### 2.1 Study design

This, prospective, observational, and analytical study was designed to establish a relationship between NfL levels in the serum as a biomarker of clinical expression of ATTRv. Participants included in this study were either presymptomatic ATTR-Val50Met mutation healthy carriers with no symptoms or signs of multisystem involvement at enrollment and adult (≥18 years) patients with symptomatic ATTRv amyloidosis who had developed polyneuropathy of different grades. The exclusion criteria comprised the inability to provide consent and/or diagnosis of any other polyneuropathy or neurological disease. All consecutive participants of the study were recruited at the outpatient clinics in real-life practice from the University Hospital Juan Ramón Jiménez, Huelva, Spain. In the present study, all patients were selected from the endemic focus of Valverde del Camino, Huelva, Spain.

All study procedures were conducted per the ethical standards as laid down in the 1964 Declaration of Helsinki and its later amendments, as well as local regulations in force. The study was granted approval by the ethics committee of the Huelva Provincial (Decision number: IB 4491/21 PI). All study participants were required to sign informed consent prior to registration.

### 2.2 Clinical evaluation

The baseline characteristics and clinical assessment of patients comprised patients’ demographic data, current ATTRv treatment, and identification of the pathogenic genetic variant in each patient. A complete clinical examination was conducted, which included an organ system-based review, the Neuropathy Impairment Score, evaluation for “red flags,” and tests such as SUDOSCAN, heart rate variability, electrocardiography, and an assessment for orthostatic hypotension among other. We did not routinely perform biopsies (e.g., labial salivary gland, skin, abdominal fat pad, or nerve) to classify carriers (26–29). This decision was based on the fact that our study subjects were from an endemic focus and often had a strong family history supporting the diagnosis.

The functional status of patients was assessed using the Polyneuropathy Disability Score (PND), as described elsewhere (30). Laboratory measurements comprised the evaluation of albumin, total protein, and serum TTR (as determined by chemiluminescence; Roche Diagnostics) in order to verify treatment adherence and disease control.

### 2.3 Blood samples

Blood was collected by standard venipuncture and processed within two hours. Immediately after collection, the samples were centrifuged at 2000 rpm for 10 min at room temperature to obtain the serum fraction. The serum was aliquoted into cryovials and stored at −80 °C in two freezers to maintain stability until further analysis.

Symptomatic patients and asymptomatic TTR gene healthy carriers were tested for NfL levels in the serum using the two available analytical platforms. In order to identify and validate the most accurate technique for NfL quantification, we employed both the NF-Light® serum enzyme-linked immunosorbent assay (ELISA) kit and the Single-molecule array (SIMoA®) NfL assay™ on the HD-X platform according to the manufacturer’s instructions. Both kits used the same capture antibodies provided by Uman Diagnostic (Quanterix®, Umea, Sweden) (31, 32). Briefly, samples were added to a 96-well ELISA plate coated with a non-competitive, monoclonal antibody enabling the binding of NfL from the sample. After two hours of incubation, the biotin-conjugated detection antibody was added, and the plate was kept at room temperature for 90 min to allow binding to occur. Streptavidin-protein horseradish peroxidase conjugate was administered after incubation, allowing the enzymatic conversion of a colorless TMB substrate to a colored product. The reaction was halted by utilizing diluted H2SO4, and the absorbance was immediately measured using an optical absorbance reader (Quanterix® NfL ELISA kit). The absorbance values were correlated to the amount of NfL in each sample by using the calibrator curve. The ELISA assay has a low limit of detection of 0.4 pg./mL and a variant coefficient ranging between <10% inter-precision and <6.6% intra-precision, respectively, as provided by the manufacturer. In addition, NfL concentration was also measured according to manufacturer’s instructions on blinded samples using the SIMoA® NF-light™ HD-X Advantage assay as described elsewhere (33). Recently, other immunoassays, such as the Electrochemiluminescence Immunoassay (ECLIA), have also been introduced for NfL detection in clinical diagnostics. Unfortunately, their commercial approval in mid-2024 occurred after the analyses for the present study had been completed (34).

In order to investigate the longitudinal in-time changes in NfL levels, five assessments of NfL levels were conducted during the study with the objective of recommending appropriate NfL cut-off values for the conversion from presymptomatic to symptomatic disease and to evaluate the changes in NfL levels during the treatment with different drugs.

### 2.4 Statistical analysis

We determined the patient sample size by utilizing data from prior research that investigated the correlation between the two NfL quantification techniques (35). To evaluate the Pearson correlation coefficient between the two assays, we implemented a two-sided Student’s t-test. Given a target power of 80% to detect a non-zero correlation (H₀: *ρ* = 0), a significance level of *p* < 0.05, and an anticipated correlation of 0.96 and a 10% dropout rate, we determined a minimum recruitment target of 15 patients per assessment.

To better mimic the representative cohort of patients with ATTRv amyloidosis from this endemic focus, the number of patients enrolled in this study was adjusted to align with the patients’ real-life demographic variations in terms of their polyneuropathy disability score (i.e., PND of 0-II). To reduce variability and ensure accuracy, the study limited the number of patients based on the analytical method, as each ELISA and SIMoA kit well plate accommodates 96 tests. After completing the control test and calibrations, a total of 75 patients were enrolled in the study and subjected to analysis.

For quantitative continuous variables, descriptive analyses were conducted using the mean and standard deviation, while categorical variables were summarized using count and percentage. We also explored and controlled the existence of outliers.

The Kolmogor-Smirnov method was employed to assess the normalcy of the quantitative variables. We used non-parametric Kruskal-Wallis tests to compare NfL levels measured by ELISA between the groups (i.e., PND of 0, I, II), followed by the Bonferroni post-hoc test. Receiver operating characteristic (ROC) curve analysis was performed to assess the ability of NfL to discriminate the transition from the presymptomatic to the symptomatic stage of the disease by plotting the true-positive fraction (or ‘sensitivity’) against the false-positive fraction (or ‘100%-specificity’) across different cut-off points. To investigate the relationship between the results of the ELISA test of each PND stage with other variables, such as sex, treatment, and SIMoA test, statistical techniques such as Pearson correlation, Student’s t-test, and analysis of variance were used. Furthermore, the area under the curve, sensitivity, and specificity of the ELISA were calculated based on the PND. In all analyses, the threshold for statistical significance was set at *p* < 0.05. All analyses were performed with SPSS version 23 and GraphPad Prism version 8.0.1.

## 3 Results

### 3.1 Patient demographics

We collected blood samples from 75 subjects of a pathogenic variant in TTR gene (Table 1). Of these, 42 patients (56.4%) were asymptomatic ATTRv healthy carriers, and 33 patients (44.0%) had clinical symptoms of polyneuropathy-associated disease, classified as PND I or PND II at the time of study entry. Thirty-three patients (44.0%) received ATTRv-specific treatments with either TTR-stabilizers (n = 13), TTR gene silencers (*n* = 15) or underwent liver transplantation (*n* = 5). A total of 42 ATTR-Val50Met mutation carriers remained untreated; of these, two patients were classified as PND I at study entry and had not yet commenced therapy.

**Table 1:** Patient demographic.

Parameter		Number of patients, *n* (%)
PND score	0	40 (53.3%)
	I	29 (38.7%)
	II	6 (8.0%)
Age, mean (standard deviation) (range)		51 (13) (22–74)
Sex	Men	36 (48.0%)
	Women	39 (52.0%)
Treatments	Not treated	42 (56%)*
	*TTR*-stabilizers	13 (17.3%)
	*TTR* gene silencers	15 (20.0%)
	Liver transplantation	5 (6.7%)

*Forty-two ATTR-Val50Met mutation carriers remained untreated. Two of these patients, who were classified as PND I at the start of the study, had not yet begun treatment.

PND, polyneuropathy disability score; TTR, transthyretin.

### 3.2 Correlations between neurofilament levels quantification using ELISA and SIMoA methodology

**Figure 1:**
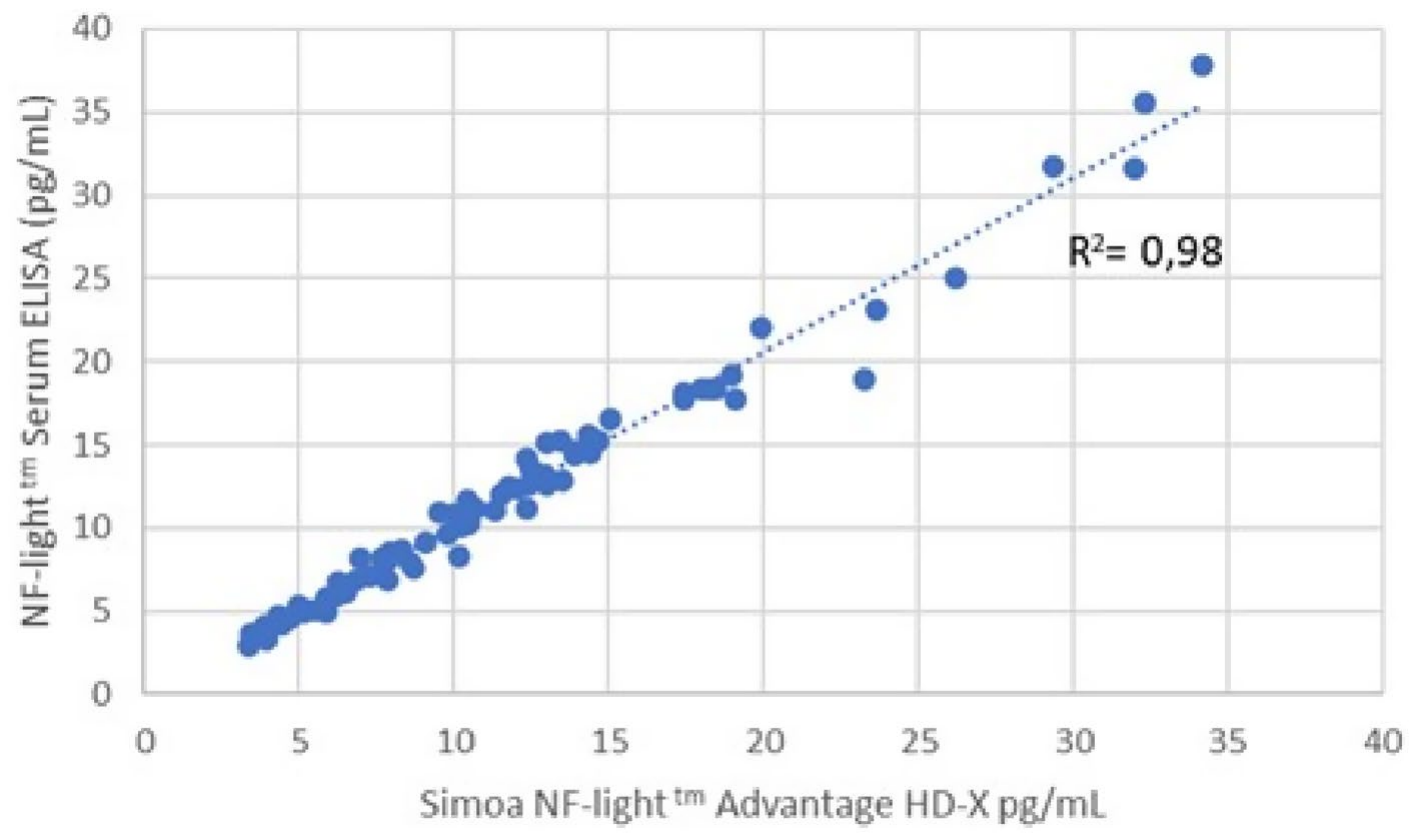
Correlation was between the SIMoA and ELISA assays. A significant correlation between ELISA and SIMoA assay was observed as both methodologies yielded almost superimposed outcomes as reported mean concentrations of NfL of 11.78 pg./mL and 11.98 pg./mL, respectively (Pearson’s *R*^2^-value = 0.9899).

Following the analysis of identical serum samples from all included patients using ELISA and SIMoA, both methodologies yielded almost superimposed outcomes as reported mean concentrations of NfL of 11.78 pg./mL and 11.98 pg./mL, respectively. Using a Pearson’s correlation analysis, wherein the ranking of values was considered, the observed R2-value was 0.9899, indicating a significant linear correlation and a favorable agreement between both methodologies (Figure 1). Although a greater dispersion was observed at higher values, the level of statistical significance *p* < 0.001 confirmed the robustness of this correlation at lowers levels of clinical interest (Supplementary Table 1). Due to a strong positive correlation between both methodologies, all further analyses were done using an ELISA assay only. Supplementary Table 2 depicts the principal performance characteristics of the main assays (36).

### 3.3 Correlation between neurofilament levels and disease stages

Serum NfL levels were statistically increased (*p* < 0.001) in symptomatic ATTRv patients with a PND I or PND II compared to ATTRv healthy carriers with a PND 0 (Table 2). The NfL increase in patients’ serum was 1.7 (PND I) to 2.6-fold (PND II) higher than in asymptomatic healthy carriers. This finding of a positive correlation is particularly intriguing, as it reinforces the efficacy of NfL levels as a reliable biomarker to distinguish the transition from the presymptomatic to the symptomatic stage of the disease.

**Table 2:** Serum neurofilament levels in different study groups.

Parameter		ELISA assay (pg/ml)		
		Mean NfL levels	Standard deviation	*p* value *
PND score	0	7.9	3.7	<0.001
	I**	13.7	4.8	
	II	20.7	8.6	
Sex	Men	13.1	8.6	0.218
	Women	10.9	6.1	
Treatments	Not treated	8.3	4.5	<0.001
	*TTR*-stabilizers	18.1	10.2	
	*TTR* gene silencers	14.8	5.0	
	Liver transplantation	18.4	8.5	

* Calculated for all levels for each parameter.

** Reported neurofilament values refer to 26 patients with PND II. Three patients with extremely high neurofilament values were excluded from the analysis as statistical outliers.

NfL, neurofilaments; PND, polyneuropathy disability score; TTR, transthyretin.

The NfL levels were significantly higher in patients on treatment compared with untreated patients (*p* < 0.001). On the other hand, we did not find any association between sex and NfL levels (Table 2). Likewise, there were no statistically significant differences between patients with symptomatic ATTRv with a PND I and those with more advanced disease with a PND II (Supplementary Table 3).

### 3.4 Neurofilament levels are a predictive biomarker of disease progression

**Figure 2:**
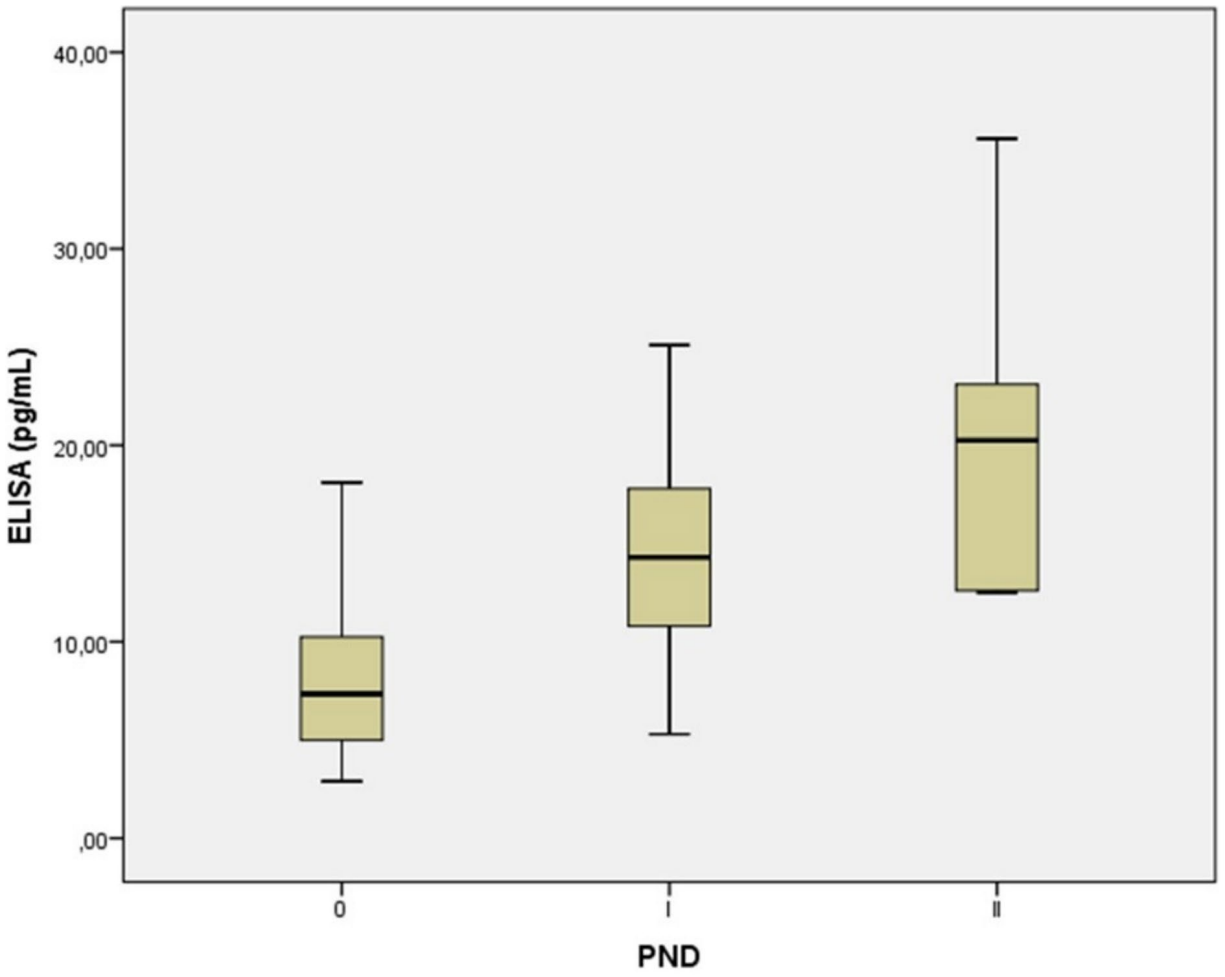
Serum neurofilament light chain concentration in in presymptomatic healthy carriers with a PND of 0, and symptomatic ATTRv patients with a PND I-II. Each box represents the area between the first (Q1) and the third (Q3) quartile (interquartile range, IQR), with the lines inside the boxes representing the mean values (Q2). A mean NfL level of over 7.9 pg./mL may signal the onset of symptoms in individuals with ATTRv, moving them from a healthy carrier state to the early stage of the disease (PND I). Levels over 20.7 pg./mL could then indicate the disease’s progression from PND I to more advanced stages (PND II and beyond). PND, polyneuropathy disability score.

We observed that the mean values of NfL levels may allow us to determine cut-off values to stratify patients according to their disease stage. Specifically, our results suggest that a mean NfL level of over 7.9 pg./mL could represent a threshold to discriminate the transition from the ATTRv healthy carriers to the symptomatic early stage of the disease (PND I), whereas those with a mean NfL level of over 20.7 pg./mL may discriminate the transition from PND I to PND II and above (Table 2 and Figure 2).

**Figure 3:**
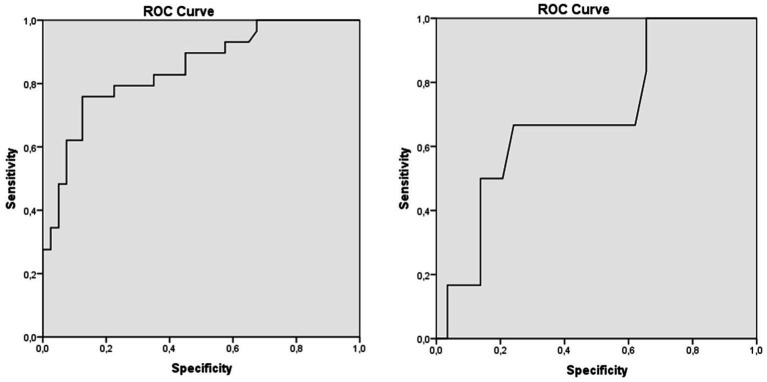
Receiver operating characteristics curves of serum neurofilaments levels. Receiver operating characteristic (ROC) curves of different thresholds of serum neurofilaments in differentiating ATTRv patients from either **(A)** presymptomatic healthy carriers (PND 0) to early-stage symptomatic (PND I), and **(B)** early-stage symptomatic (PND I) to patients with motor dysfunctions (PND II). A < 7.9 pg./mL NfL concentration distinguished healthy ATTRv healthy carriers from PND I patients with good accuracy (AUC 0.847; sensitivity 90.0%, specificity 55%). An NfL level of 18.4 pg./mL showed moderate accuracy for differentiating between PND I and PND II patients (AUC 0.695; sensitivity 67.0%, specificity 86%).

In addition, the AUC of the ROC curve comparing ATTRv healthy carriers with symptomatic ATTRv patients with a PND I was 0.847 (95% confidence interval [CI]: 0.940–0.754; *p* < 0.001). We found that an NfL concentration of <7.9 pg./mL could discriminate between these two groups with a sensitivity of 90.0% and a specificity of 55% (Figure 3A). When comparing early symptomatic patients with a PND I with patients presenting symptoms and signs of motor dysfunction with a PND II, we found an AUC of 0.695 (95% CI, 0.923–0.468, *p* < 0.001) and the NfL level of 18.4 pg./mL may discriminate these patients with a sensitivity of 67.0% and a specificity of 86% (Figure 3B).

### 3.5 Analysis of neurofilament levels according to treatment

Patients on treatment with TTR gene silencers, as the most potent drugs for this disease capable of blocking disease progression, had the lowest mean NfL levels (14.8 pg./mL) compared to patients who had received a treatment with either TTR-stabilizers (18.1 pg./mL) or underwent liver transplantation (18.4 pg./mL) (Table 2).

Upon conducting an analysis using Bonferroni’s *post hoc* test for multiple comparisons among patient groups, we observed that patients treated with TTR gene silencers showed a clear trend towards lower mean levels of NfL compared with those treated with TTR-stabilizers or liver transplantation (Table 3). For instance, when comparing patients with a PND I who received TTR-stabilizers with those treated with TTR gene silencers, their NfL levels were considerably higher (16.6 versus 13.3 pg./mL). This difference was especially evident in patients with a PND II, where TTR-stabilizers had a much lower effectiveness in reducing NfL levels than treatment with TTR gene silencers (35.6 versus 17.7 pg./mL) (Table 3).

**Table 3:** Analysis of neurofilament levels and disease severity according to treatment.

Bonferroni’s *post hoc* test			ELISA assay (pg/ml)			
Treatments	PND stage		Number of patients	Mean	SD	*p* value*
Not treated	PND	0	40	7,9	3,73	0,351
		I	2	16.0	12,94	
		II	0	-	-	
*TTR*-stabilizers	PND	0	0	-	-	0,308
		I	12	16.6	9,16	
		II	1	35.6	-	
*TTR* gene silencers	PND	0	0	-	-	0,206
		I	10	13,3	4,57	
		II	5	17,7	5,05	
Liver transplantation	PND	0	0	-	-	
		I	5	18,4	8,54	
		II	0	-	-	

*Calculated for all levels for each parameter.

SD, standard deviation; PND, polyneuropathy disability score; TTR, transthyretin.

## 4 Discussion

The results of this study confirm the usefulness of analyzing serum NfL in patients with ATTRv as a reliable, reproducible, and non-invasive biomarker. Given that in healthy individuals, only insignificant levels of NfL can be observed, increased NfL may represent a surrogate biomarker for screening individuals with positive family history and for early diagnosis of ATTRv in patients still without symptoms as well as for the monitoring of disease progression and response to treatment (4, 20). Those findings from our study are in line with a recent review of 13 articles reporting that NfL is an early and sensitive process biomarker for neuropathy in systemic amyloidosis with potential to be used both for early detection of neuropathy and for disease progression in patients with systemic amyloidosis (23). Noteworthy, our study has demonstrated that both ELISA and SIMoA are reliable methods for NfL quantification, as the results of the present study demonstrate a strong correlation between the concentrations of NfL in serum obtained after comparing ELISA and SIMoA methodologies, with an R2-value of 0.98 and *p* < 0.001 (Figure 1). The highly sensitive SIMoA NfL assay was previously the method of choice for early disease stages due to its very low lower detection limit. This sensitivity allowed for the detection of minimal changes in NfL values, which improved diagnostic accuracy. Since recently, second-generation ELISA tests (Quanterix) have matched the detection limits and diagnostic sensitivity of the SIMoA (Quanterix) assay in the initial stages (see Supplementary Table 2). As a result, both methods are now comparable and interchangeable for practical purposes (Figure 1) and the choice between the two depends on the availability and operational needs of each laboratory. Other studies have also demonstrated that ELISA and SIMoA assays are equivalent for the evaluation of NfL concentration and can be used in routine clinical practice for longitudinal follow-up (37, 38). Recent reports indicate that the new electrochemiluminescence immunoassay such as Siemens ADVIA Centaur® XP, has a high correlation between NfL concentrations in serum (*R*^2^ = 0.907), as observed using the SIMoA array (Supplementary Table 2) (36). In addition, a recent commutability study that analyzed the NfL concentration in blood using four different analytical platforms such as SIMoA, OLINK, Ella, and Atellica® Solution, also reported a high correlation between the different platforms (Spearman’s *p* ≥ 0.96) (35). In contrast, other groups reported a much weaker correlation between both methods in patients with multiple sclerosis using both cerebrospinal fluid and serum samples (39, 40). In addition, in the recent review, a good correlation was reported between the outcomes of the Simoa and Ella assays. However, no such correlation was observed with the first-generation ELISA used in one of the studies. This discrepancy is likely attributable to its higher lower limit of detection (33 pg./mL), which was insufficient to detect early neuronal damage in asymptomatic TTRv healthy carriers who progress to symptomatic disease (23). Indeed, as per correlation analysis, we observed a somewhat greater dispersion at higher values, probably owing to the detection limits of the technique, with upper and lower limits of quantification of 400 pg./mL and 0.8 pg./mL, respectively. Furthermore, the calibrators we used for both methods did not reach such high values, which may have contributed to this dispersion. Despite this, the main focus of the correlation analysis was to ensure linearity at lower values, specifically in the range of 0.8 pg./mL to 40 pg./mL, since our primary goal was to accurately detect the onset of the disease in early stages, thereby enabling timely treatment initiation and improving patient prognosis.

Additionally, we sought to demonstrate a real-life association between NfL levels and polyneuropathy impairment scores of disease severity by means of PND. We observed significant elevations in NfL levels in patients with early-stage ATTRv amyloidosis (PND I) compared to ATTRv healthy carriers (PND 0) (Table 2). This suggests that for asymptomatic TTR carriers who are clinically indistinguishable, a specific NfL threshold might serve as a biomarker for disease conversion, providing a potential cutoff value to help distinguish between the presymptomatic stage and the onset of clinically observed sensory or sensorimotor neuropathy. Our results align with previous studies that reported three to ten times higher serum NfL levels in ATTRv amyloidosis patients compared to ATTRv healthy carriers and healthy controls (2, 4, 20, 22–24). However, in our study we did not observe statistically significant differences between patients with symptomatic ATTRv with a PND I versus those with a PND II with more advanced disease and greater axonal loss and sensorimotor neuropathy. This result is likely due to the limited number of samples taken from patients with a PND II (*n* = 6). Yet, other studies have reported a significant correlation between NfL levels and disease stage (4, 22, 24). We also observed that NfL levels were significantly higher in treated patients than in untreated patients with a PND 0 (Tables 2, 3). This outcome was expected, as patients who do not receive treatment are often ATTRv healthy carriers with very low NfL values. Other groups have also observed that despite the fact that patients with symptomatic ATTRv receive active treatment, their NfL levels decrease but do not normalize and rarely return to baseline values, remaining significantly higher than those of TTR healthy carriers (2, 21, 41). In our study, the administration of TTR gene silencing therapies has proven to be highly effective in reducing NfL levels in advanced stages of disease with a PND II (Table 3). This discovery might hold significant clinical implication, as it suggests that treatments are effective in reducing neurofilaments, thereby slowing down the progression of the disease.

Despite the limited number of patients enrolled in this study, our data confirm that, during treatment with a TTR-stabilizer, NfL levels remain stable or increase, requiring urgent switching to a TTR-gene-silencing treatment upon the first signs of rising NfL levels. After treatment with a TTR-gene silencer, the levels of NfL decreased or were stable, and this decrease was sustained with extended treatment. This further validates the utilization of NfL as a valuable instrument for monitoring treatment response. Further studies are warranted to provide additional evidence to support the hypothesis that treating even asymptomatic patients may prove beneficial, as it may reduce indicators of neuronal damage and potentially delay the onset of clinical symptoms.

In agreement with previous studies, we found no differences in NfL levels between the sexes (11). Our prior data from a larger cohort of ATTRv patients from the same endemic focus of Valverde del Camino, Huelva have demonstrated that a higher proportion of men developed symptoms than women, possibly due to the presence of polymorphisms in the TTR gene that modulate disease expression and clinical symptoms. Furthermore, the results also indicated that patients over 70 years had a relatively low overall penetration of 51% (42). These data further underscore the complexity of monitoring asymptomatic healthy carriers according to their ages and sex, and emphasize the use of NfL as a non-invasive biomarker compared to the presently used painful and time-consuming electromyography studies. Other studies have also revealed a significant positive correlation between patients’ age and renal function (measured as estimated glomerular filtration rate) or a negative correlation between patients’ body mass index and NfL levels (4, 11). However, Maia et al. (4) reported that the NfL increase in plasma was primarily due to the disease stage, outweighing the age-related effects. In addition, a significant age-related increase in NfL, combined with the cut-off’s modest 55% specificity, raises questions about its effectiveness for classifying all healthy ATTRv carriers. While we use age-adjusted percentiles from the Swiss multiple sclerosis group in clinical practice, our study’s limited sample size prevented us from drawing definitive conclusions in specific age subgroups (e.g., 50–55, 55–60, and >60) (43).

The ROC curves and the corresponding AUC show that NfL levels have a good predictive ability to distinguish patients with symptomatic disease from healthy carriers and different stages of symptomatic amyloidosis. Our study showed that an NfL level of 7.9 pg./mL might help discriminate between presymptomatic and symptomatic individuals with extremely high diagnostic accuracy, a sensitivity of approximately 90%, and a specificity of 55%, suggesting that NfL may play a significant role in recognizing the onset of symptomatic disease. Nonetheless, the precise threshold of NfL levels to predict the transition from asymptomatic to symptomatic ATTRv patients has yet to be established, and it has been observed that it ranges from 10.6 pg./mL to 37.0 pg./mL (4, 9, 20–22). Undoubtedly, the utilization of various methodologies for NfL quantification with varying analytical sensitivity levels and different samples (such as serum, plasma, or cerebrospinal fluid) may cause some disparities in cutoff values. Other studies have suggested that, instead of using a single NfL cutoff for all patients, individual cutoff values should be used to accurately predict the transition from asymptomatic to symptomatic disease (22). To distinguish patients with a PND I with sensory neuropathy from those with a PND II with motor neuropathy, cutoff values of 66.9 pg./mL (sensitivity 61.5% and specificity 92.3%) or 75.7 pg./mL (sensitivity 84.6% and specificity 80%) were proposed in both early- and later-onset cohorts, respectively (4). These findings are much higher than those observed in our study (i.e., 18.4 pg./mL) most likely because Maia et al. evaluated patients with more advanced stages of ATTRv and with very high NfL levels at baseline (4).

Our study holds some limitations, principally considering the limited number of samples, which reduced the power of our analyses and prevented us from expressing the percentage of NfL increase adjusted for age. Another important limitation is the lack of a long-term longitudinal follow-up to confirm the role of NfL as a marker of disease progression and response to therapy. Currently, most studies have enrolled very heterogeneous individuals with ATTRv to create different cohorts. Yet, in more homogeneous cohorts of patients the cutoff values of NfL will be much more realistic and representative for these groups of patients.

In conclusion, this study suggests that serum NfL may be a valuable biomarker in a real-world ATTRv population for early diagnosis, monitoring disease progression, and assessing treatment response. Furthermore, our results also suggest potential cutoff values for predicting the transition from asymptomatic to symptomatic disease and from the early-to-late stage of disease, which could facilitate improved patient stratification and disease management.

## Data Availability

The raw data supporting the conclusions of this article will be made available by the authors, without undue reservation.
