# Supplementary material for: Clinical utility of neurofilament light chain as a biomarker for disease onset and progression in hereditary transthyretin amyloidosis
Source: Front Neurol. 2025 Oct 8;16:1660344. doi: 10.3389/fneur.2025.1660344 (PMC12540126; doi:10.3389/fneur.2025.1660344)
Supplement: Supplementary file 1 [file Data_Sheet_1.pdf]

Supplementary Material

1 Supplementary Tables

Supplementary table 1. Pearson correlation analysis between the SIMoA method and ELISA

**Methods**		SIMoA (pg/ml)	ELISA (pg/ml)
**SIMoA (pg/ml)**	Pearson's correlation	1	.990*
	Significant (two-tailed)	-	0.000
	Number of patients	75	75
**ELISA (pg/ml)**	Pearson's correlation	.990*	1
	Significant (two-tailed)	0.000	-
	Number of patients	75	75

* Significance level defined as $p<0.01$ (two-tailed).

Supplementary table 2. Information about the assays

Parameter	ELISA (Quanterix)	SIMoA (Quanterix)	ADVIA Centaur® XP immunoassay (Siemens)
**Limit of detection**	0.4 pg/ml	0.026-0.162 pg/ml	0.71 pg/mL
**Limit of quantification**	0.8 pg/ml	0.53 pg/ml	3.86 pg/ml
**Range of linearity**	0.5-400 pg/ml	0.4-360 pg/ml	3.86-500 pg/ml
**Correlation versus SIMoA**	0.9899 (this study)	-	0.907 (26)<br>0.97 (29)
**Capture antibody provider**	Uman Diagnostics/Quanterix®	Uman Diagnostics/Quanterix®	Uman Diagnostics/Quanterix®
**Routine applicability**	Kits of 96 tests, requires extra equipment in the laboratory, yields 44 samples per plate.	Kits of 96 tests, requires extra equipment in the laboratory, yields 44 samples per plate.	Individual test, no need for extra equipment since can be done with the same equipment used in routine laboratory work.<br>Not limited test but requires quality control at each use.
**Costs**	Average cost, to optimize it is needed to mount the complete plate.	Average cost, to optimize it is needed to mount the complete plate.	High-cost but facilitates the optimization of resources since the determinations can be made individually.

Supplementary table 3. Comparison of neurofilament levels according to the polyneuropathy disability score (PND)

**Polyneuropathy disability score** **Bonferroni's post hoc test**		**Difference of median** **Neurofilament light chain levels (NfL)**	**Standard deviation**	***p* value**	**Confidence interval, 95 %**	
					**Lower limit**	**Upper limit**
**0**	I	-7.80158*	1.45866	0.000	-11.3771	-4.2261
	II	-12.77342*	2.61838	0.000	-19.1916	-6.3552
**1**	0	7.80158*	1.45866	0.000	4.2261	11.3771
	II	-4.97184	2.68237	0.204	-11.5469	1.6032
**2**	0	12.77342*	2.61838	0.000	6.3552	19.1916
	I	4.97184	2.68237	0.204	-1.6032	11.5469

*Significance difference between median values defined as $p<0.05$.
